# Discriminatory Experiences Among Black Youth: How Encounters and Expectations Explain Emotional Well-Being

**DOI:** 10.1007/s11121-023-01540-2

**Published:** 2023-06-17

**Authors:** Nicole M. Summers-Gabr, Mikiko Sato, Sarah M. Chilenski, Francisco Villarruel, Paula Smith, Charles Henderson, Jeremiah Newell, Hilder Wilson, Astrid Craig

**Affiliations:** 1 Department of Population Science and Policy, Southern Illinois University School of Medicine, 201 E. Madison, Springfield, IL 62702 USA; 2 Department of Human Development and Family Studies, Michigan State University, East Lansing, USA; 3 Edna Bennett Pierce Prevention Research Center, Pennsylvania State University, State College, USA; 4 Department of Educational Leadership and Policy, University of Utah, Salt Lake City, USA; 5 University Medical Billing, University of Utah, Salt Lake City, USA; 6 Mobile Area Education Foundation, Mobile, USA; 7 Dallas County Juvenile Probation, Selma, USA

**Keywords:** Discrimination, Black youth, Adolescence, Social-emotion development, Mental health

## Abstract

Research suggests that encounters with racism are related to depression in Black youth. However, less is known about how experienced racial discrimination can influence other aspects of well-being among Black youth including their socio-emotional development and behavior. In addition, emerging literature highlights the critical ways anticipated racial discrimination may impact the emotional well-being of Black youth. To address these gaps, the current study assessed whether experienced discrimination was associated with higher levels of internalizing problems (anxiety/depression, suicidal thoughts) and lower levels of socio-emotional development (emotion regulation, prosocial behavior). We then tested whether expected discrimination contributed to similar patterns. Lastly, this study examined how age and gender moderated this relationship. Across eight schools in three communities, 1435 Black youth (56.57% female; 56.40% 10th grade) in 10th and 12th grades responded to the Youth Experience Survey. Using a series of hierarchical linear and hierarchical binary logistic regressions, results found that those who experienced racial discrimination and expected discrimination demonstrated higher internalizing problems and lower socio-emotional development; however, expected discrimination often accounted for more variance than experienced. These findings suggest the multifaceted influence both experienced and expected racial discrimination have on the well-being of Black youth and can provide important insights to community prevention systems.

## Introduction

The impact of systemic racism has been at the forefront of national discourse and public health, particularly for Black youth. There is a critical need to understand the experiences of Black youth and how systemic barriers affect their emotional well-being. Racism can have dire consequences on the mental health of youth (Kirkinis et al., 2018; Walker et al., 2017). As racial inequality continues, scholars are responsible for documenting the mental health disparities that affect Black youth. This study provides details about mental health and specific important mental health-related processes thought to be impacted by discrimination. Therefore, the first aim of this investigation was to examine whether experienced and anticipated racial discrimination were associated with high levels of internalizing problems, specifically depression/anxiety and suicidal ideation. The second aim was to explore the effect experienced and expected racial discrimination had on social-emotional development, specifically emotion regulation and prosocial behavior. Lastly, this study explored how age and gender moderated the relationship between experienced and anticipated discrimination and the aforementioned outcomes.

### Perceived Racial Discrimination and Mental Health

Perceived racial discrimination, which is biased and unjust treatment based on one’s racial identity (Assari et al., 2019), can result in poor mental and physical health outcomes for Black youth (Kirkinis et al., 2018). More specifically, experienced discrimination has been associated with depressive symptoms (Seaton & Yip, 2009), decreased self-esteem (Seaton et al., 2010), and increased suicidal thoughts (Walker et al., 2014, 2017). Studies have shown the widespread prevalence of racial discrimination among Black youth; reports typically increased during middle and late adolescence (Umaña-Taylor, 2016). For instance, research suggests that during early adolescence anywhere from 10% (Nagata et al., 2021) to 38% (Roberts et al., 2012) of Black youth experienced at least some racial discrimination. However, during mid to late adolescence, the proportions were as low as 22% (McNeil Smith & Fincham, 2015), but leaned more toward higher proportions such as 62.5% (Sellers et al., 2006) or even 89% of youth (Roberts et al., 2012). The complexity in these issues is furthered by youths’ ability to perceive discrimination (Cunningham et al., 2019) and the increase in stereotyping of African Americans by White and Black youth (Copping et al., 2013) during adolescence. Therefore, both mental health problems as well as the ability to perceive racial aggressions increase during the same developmental period and likely reinforce each other, which ultimately further exacerbates mental health disparities in adulthood due to its cumulative consequences.

Despite the well-established relationship between discrimination and mental health, there are relatively few studies on the influence discrimination has on social-emotional development, such as emotion regulation and prosocial behavior (Riley et al., 2021). Because discrimination is an emotion-eliciting experience, it compels people who have experienced it to be adaptive (Riley et al., 2021). One challenge in parsing out the impact of racial discrimination on social-emotional development is that it can be considered both an outcome (e.g., Padilla‐Walker et al., 2012) and a protective factor (Raposa et al., 2016; Taylor et al., 2017; Troy & Mauss, 2011). For instance, in the context of discrimination and emotion regulation, that could mean that the better someone’s emotion control is, the less likely there is to have suicidal ideation in response to experiencing discrimination. However, that could also mean that the more discrimination someone experiences, the harder it is for an individual to control emotions like anger, sadness, or fear.

Understanding the direction of the relationship between discrimination and prosocial behaviors is also challenging to deconstruct. Prosocial behaviors are what foster positive relationships and without them could lead to marginalization and social exclusion (Davis & Carlo, 2019). In one study, Lozada et al. (2017) found that school-based discrimination actually increased prosocial behaviors in Black male adolescents and theorized it was because there is an expectation for youth to organize and act in their community. In another study, but with Latino youth, Davis et al. (2021) found that the direction of the relationship between discrimination and prosocial behavior depended on whether prosocial behaviors were conducted publicly or altruistically (Davis et al., 2021). As research continues to progress in this topic, it is important to continue considering the relationship between social-emotional development, such as prosocial development, and poor mental health outcomes because it is crucial to understand how discrimination impacts the whole child (Davis et al., 2021).

### Anticipated Racial Discrimination and Well-Being

Extensive literature underpins the impact of experienced discrimination on mental health; however, less is known about how anticipated discrimination influences well-being. Anticipated or expectations of racial discrimination is the notion that one will be discriminated against because of one’s racial background (Herda, 2016). Anticipated racial discrimination derives from cultural racism. Williams et al. (2019) described cultural racism as “the instillation of the ideology of inferiority in the values, language, imagery, symbols, and unstated assumptions of the larger society” (p. 110). Furthermore, cultural racism constitutes a society where systemic racism reinforces macro- and micro-levels of discrimination. In other words, cultural racism may generate beliefs among people of color that magnify chronic fear, anticipation, and stress of societal prejudice and discrimination because of one’s racial identity. Scholars argue that in addition to experienced discrimination, more empirical studies on anticipated discrimination are needed to better understand the link between discrimination and well-being (Herda, 2016).

Recent studies indicated that anticipated racial discrimination has important implications for youth mental well-being. For instance, Zimmerman and Miller-Smith (2021) explored the relevance of anticipated racial discrimination for depression and suicidal behavior among a diverse youth sample from the *Project on Human Development in Chicago Neighborhoods*. They found that anticipated racial discrimination was positively associated with major depressive disorder and suicidal behavior. Additionally, Black and Hispanic youth were disproportionately exposed to racial discrimination, with Black youth reporting the highest level of experienced and anticipated discrimination followed by Hispanic youth. These findings suggest that anticipated racial discrimination is highly relevant for Black youth, and research needs to understand the effect it has on other dimensions of well-being in addition to depression and suicidality.

### Present Study

The purpose of this study is to examine the relevance of discrimination to internalizing behaviors, including anxiety/depression, suicidal thoughts, and social-emotional development, specifically emotion regulation, and prosocial behavior. In addition, while research has been conducted predominantly in large metropolitan Black-majority communities (e.g., Seaton & Yip, 2009), less empirical data is available on Black youth living in less densely populated areas. To fill this gap, this study utilizes data from Black youth that lived in small cities and rural areas.

The current study assessed how discrimination experienced in the past year was associated with higher levels of internalizing problems, specifically anxiety/depression and suicidal thoughts, and lower levels of social-emotional development, including emotion regulation and prosocial behavior. Next, we tested whether anticipation of discrimination contributed to similar patterns among Black youth. Finally, we tested whether discriminatory experiences or expectations had independent effects on youth outcomes.

## Method

The data for this project came from three communities across two southern USA counties involved in implementing a community-systems intervention called Evidence2Success®. The Evidence2Success framework is a service-delivery system that brings together stakeholders in systems and communities to select and integrate tested and effective programs into communities based on youth-reported risk and protective factors, and outcomes (Annie E. Casey Foundation, n.d.). The current study does not focus on the implementation of the Evidence2Success framework, but rather used secondary data analysis on the youth assessment to explore the stated research questions. The unique census sample of 10th- and 12th-grade Black youth, with the combination of data needed to address these research questions, positioned us to examine our research questions. The analysis, no. 19–531, was given Non-Human Subject Research Determination by Southern Illinois University School of Medicine.

### Participants

Youth (*n* = 1,860 students) in 10th and 12th grades responded to the Youth Experience Survey. Only the data from youth who identified as Black/African American were included in the analysis. Specifically, youth were asked, “Please choose the ONE answer that BEST describes what you consider yourself to be: (a) White, (b) Black, African American or African, (c) Hispanic, Latino or Spanish origin, (d) American Indian or Alaska Native, (e) Asian, (f) Native Hawaiian or Pacific Islander, (g) Multiracial or biracial, or (h) Other.” Across eight schools, 1435 Black youth or 70.15% of the total sample (56.57% female; 56.40% 10th grade) 10th and 12th grades responded to the Youth Experience Survey.

### Measures

#### Control Variables

Site location was entered into step 1 as a covariate.

#### Independent Variables

Three variables were included in step 2 for each analysis: age, gender, and racial discrimination (experienced or expected or both). See Table 1 for descriptive statistics.

**Table 1:** Correlation matrix of independent, dependent, and control variables

Variable	*N*	M	SD	3	4	5	6	7	8	9	10
3. Age (centered)	1155	-	-	-	-	-	-	-	-	-	-
4. Gender (female)	1152	-	-	−.01	-	-	-	-	-	-	-
5. Experienced discrimination (centered)	1155	1.31	1.54	.10***	−.07*	-	-	-	-	-	-
6. Expected discrimination (centered)	1149	1.38	1.09	.05	−.07*	.39***	-	-	-	-	-
7. Anxiety/depression	1132	3.26	2.69	−.04	.34***	.05	.04	-	-	-	-
8. Suicidal thoughts	524	17.94****	-	.04	.09	.11	.19***	.35***	-	-	-
9. Prosocial behavior	1135	6.79	2.53	.02	.20***	−.06*	−.10**	.16***	−.02	-	-
10. Emotion regulation	1132	6.96	2.99	.06*	−.16***	−.09**	−.12***	−.28***	−.21***	.22***	-

Out of 524 youth, 94 (17.94%) reported suicidal thoughts in the past 12 months. The sample size for this item is lower than the others because one community excluded the item from their survey

* < .05; ** < .01; *** < .001. ****

##### Experienced Discrimination

Two items examined first-hand recollections of racial discrimination in the past 12 months (*r* = 0.39): (a) “In the past year (12 months); how often have you been treated badly by other people because of your race?” (b) “In the past year (12 months), how much negative discrimination have you experienced?” Items were rated on a 4-point scale (i.e., 0 = never; 3 = often). These two items were originally part of a 3-item scale on racism and discrimination designed by the Social Development Research Group for the Seattle Social Development Project Surveys. However, these were separated out by the current authors to form an “experienced discrimination” scale and an item about “expectations of discrimination” due to differences in time where discrimination either took place in the past year or was anticipated.

##### Expected Discrimination

One item on a 4-point scale assessed how much youth thought their race may affect their prospects, “Do you think it will be harder for you to get ahead in life because of your race?” from 0 (not at all harder) to 3 (a lot harder).

##### Gender

Gender was coded as a binary variable (0 = male; and 1 = female).

##### Age

Age was a continuous variable in years.

#### Dependent Variables

Four dependent variables were included in the analysis: (a) anxiety/depression, (b) suicidal thoughts, (c) prosocial behavior, (d) and emotion regulation.

##### Anxiety/Depression

The Anxiety/Depression scale was originally the full Emotional Symptoms 5-item subscale from the Strengths and Difficulties Questionnaire. It was renamed in Evidence2Success to capture anxiety and depression. Students were given statements and asked to rank how true the manifestations of anxiety and depression were within the last 12 months (*a* = 0.80). Responses ranged on a 3-point scale from 0 (Not true) to 2 (Certainly). The language in the statements was as follows: (a) I get a lot of headaches, stomachaches, or sickness; (b) I worry a lot; (c) I am often unhappy, depressed, or tearful; (d) I am nervous in new situations, I easily lose confidence; and (e) I have many fears, I am easily scared.

##### Suicidal Thoughts

The item was based on the 2011 Youth Risk and Behavioral Surveillance Survey from the Center for Disease Control. The original item read “During the past 12 months, did you ever seriously consider attempting suicide?” The item was modified to “During the past year (12 months), have you seriously thought about killing yourself? And better fit with the question leads to the rest of the survey. Students responded with a “yes” or “no.”

##### Prosocial Behavior

The entire 5-item subscale “prosocial behavior” came from the Strengths and Difficulties Questionnaire (*a* = 0.82): (a) I try to be nice to other people. I care about their feelings; (b) I usually share with others, (c) I am helpful if someone is hurt, upset, or feeling ill; (d) I am kind to younger children; and (e) I often volunteer to help others (parents, teachers, children). Students rated items on a 3-point scale from 0 (Not true) to 2 (Certainly true).

##### Emotion Regulation

Finally, the emotional regulation scale is derived from the International Youth Development Study (2003) developed by the Social Development Research Group. Students reported their level of agreement to four statements about their perceived ability to control their emotions (*a* = 0.80). Items were rated on a 4-point scale from 0 (No!) to 3 (Yes!). The four statements were as follows: (1) “I know how to relax when I feel tense”; (2) “I am always able to keep my feelings under control”; (3) “I know how to calm down when I am feeling nervous”; and (4) “I control my temper when people are angry with me.”

### Procedure

Guardians were informed about the survey and had the option to abstain from participation. Students in 10th grade and 12th grade across eight schools were administered the Youth Experience Survey during the school day in a single testing session. For one testing site, data collection was in the fall, and for the other two sites, data collection was in the spring. The schools had a majority black student population (*M* = 88.19%, *SD* = 18.17%) which ranged from 56.00 to 100% Black. Students did not receive any direct benefits for their participation.

#### Data Integrity

Data were examined using IBM SPSS Statistics 28.0.0.0. Before data analysis began, data were inspected for the integrity of responses. The youth responded to an item that was used as a check for honesty: “how honest were you in filling out this survey?” Responses ranged from 1 (very honest) to 5 (not honest at all). Only data from youth who responded 1(very honest) or 2 (pretty honest) was retained for the final analysis. Of the total participants, 12.06% (173 people) did not complete the item, and 7.37% (106) people were filtered out because they selected 3 (honest sometimes) or 4 (honest occasionally). This left a total sample size of (*n* = 1156, 56.6% female). The respondents in the final dataset were *M* = 16.57 (*SD* = 1.18) years old.

### Plan of Analyses

Three models were created for each outcome of the four outcomes: internalizing problems (anxiety/depression, suicidal thoughts) and social-emotional development (prosocial behavior, emotion regulation). A series of either hierarchical linear or hierarchical logistic regressions were performed where step 1 controlled for site and step 2 tested how *age*, *gender*, *experienced discrimination,* and the two interaction terms were associated with internalizing problems (anxiety/depression, suicidal thoughts) and social-emotional development (prosocial behavior, emotion regulation). Then another series of analyses were performed to test how after controlling for site, how *age*, gender, *expected discrimination*, and its *interaction terms* also contributed to the same outcomes. Finally, another series used the same models but combined *experienced* and *expected discrimination* together in the same step to test whether one form of discrimination contributed more to each of the four outcomes than the other. See Table 2 for analyses on experienced discrimination, Table 3 for analyses of expected discrimination experiences, and Table 4 for the combined model.

**Table 2:** Hierarchical multiple regressions and hierarchical logistic regression models for experienced discrimination

Scale	Variable	Cumulative		Simultaneous				
		*R*^2^ change	Sig. *F* change	*B*	SE	*p* value	lower CI (95%)	upper CI (95%)
Anxiety/Depression	Step 1	.00	2.104					
	Site A							
	Site B							
	Step 2	.12	< .001					
	Age			−0.07	0.07	.264	−0.20	0.06
	Gender			1.86	0.15	< .001	1.56	2.15
	Experienced discrimination			0.12	0.05	.031	0.03	0.22
Suicidal Thoughts	Step 1							
	Site A	N/A	N/A					
	Site B							
	Step 2							
	Age			0.11	.10	.274	0.91	1.37
	Gender			0.55	.24	.023	1.08	2.78
	Experienced discrimination			0.19	.07	.005	1.06	1.39
Prosocial Behavior	Step 1	.00	.867					
	Site A							
	Site B							
	Step 2	.04	< .001					
	Age			0.06	.07	.395	−0.07	0.18
	Gender			1.06	.15	< .001	0.72	1.31
	Experienced discrimination			−0.08	.05	.090	−0.18	0.01
Emotion Regulation	Step 1	.00	.678					
	Site A							
	Site B							
	Step 2	.04	< .001					
	Age			0.17	.08	.023	−0.02	0.32
	Gender			−1.01	.18	< .001	−1.36	−0.66
	Experienced discrimination			−0.20	.06	< .001	−0.31	−0.08

Age and Discrimination are centered. Suicidal Thoughts does not have a R^2^ change or F change because a hierarchical logistic regression was tested

**Table 3:** Hierarchical multiple regressions and hierarchical logistic regression models for expected discrimination

Scale	Variable	Cumulative		Simultaneous				
		*R*^2^ change	Sig. *F* change	*B*	SE	*p* value	Lower CI	Upper CI
Anxiety/Depression	Step 1	.00	.125					
	Site A							
	Site B							
	Step 2	.12	< .001					
	Age			−0.06	.07	.340	−0.19	0.07
	Gender			1.86	.15	< .001	1.56	2.16
	Expected discrimination			0.15	.07	.036	0.01	0.28
Suicidal Thoughts	Step 1							
	Site A	N/A	N/A					
	Site B							
	Step 2							
	Age			0.08	.10	.466	0.88	1.32
	Gender			0.59	.24	.016	1.11	2.90
	Expected discrimination			0.48	.11	< .001	1.30	2.02
Prosocial Behavior	Step 1	.00	.871					
	Site A							
	Site B							
	Step 2	.05	< .001					
	Age			0.05	.06	.426	−0.08	0.17
	Gender			0.99	.15	< .001	0.69	1.28
	Expected discrimination			−0.20	.07	.004	−0.33	−0.61
Emotion Regulation	Step 1	.00	.691					
	Site A							
	Site B							
	Step 2	.05	< .001					
	Age			0.15	.76	.047	0.00	0.30
	Gender			−1.06	.18	< .001	−1.41	−0.72
	Expected discrimination			−0.37	.08	< .001	−0.53	−0.21

Age and expected discrimination are centered. Suicidal Thoughts does not have an *R*^2^ change or *F* change because a hierarchical logistic regression was tested

**Table 4:** Hierarchical multiple regressions and hierarchical logistic regression models for both discrimination types

Scale	Variable	Cumulative		Simultaneous				
		*R*^2^ change	Sig. *F* change	*B*	SE	*p* value	Lower CI (95%)	Upper CI (95%)
Anxiety/Depression	Step 1	.00	.125					
	Site A							
	Site B							
	Step 2	.12	< .001					
	Age			−0.07	.07	.269	−0.20	0.06
	Gender			1.87	.15	< .001	1.57	2.15
	Experienced			0.09	.05	.095	−0.02	0.19
	Expected			0.10	.08	.186	−0.05	0.25
Suicidal Thoughts	Step 1							
	Site A	N/A	N/A					
	Site B							
	Step 2							
	Age			0.07	.11	.486	0.88	1.32
	Gender			0.61	.25	.013	1.14	2.98
	Experienced			0.09	.08	.231	0.94	1.27
	Expected			0.43	.12	< .001	1.22	1.95
Prosocial Behavior	Step 1	.00	.871					
	Site A							
	Site B							
	Step 2	.05	< .001					
	Age			0.06	.07	.387	−0.07	0.18
	Gender			0.98	.15	< .001	0.69	1.28
	Experienced			−0.04	.05	.402	−0.15	0.06
	Expected			−0.17	.07	.020	−.032	−0.03
Emotion Regulation	Step 1	.00	.691					
	Site A							
	Site B							
	Step 2	.05	< .001					
	Age			0.17	.08	.029	0.017	0.32
	Gender			−1.08	.18	< .001	−1.43	−0.73
	Experienced			−0.13	.06	.034	−0.25	−0.01
	Expected			−0.30	.09	< .001	−0.47	−0.13

Age and discrimination are centered. Suicidal Thoughts does not have an *R*^2^ change or *F* change because a hierarchical logistic regression was tested

Participants’ experiences with racism were lower than what has been reported in other studies (Appendix). While 71.11% expected their race to make it harder to get ahead in life, 31.57% said they were treated badly because of their race, and 45.10% said they experienced negative discrimination. Descriptive statistics for all measures are presented in Table 1. Bivariate correlations investigated the simple associations among variables, including whether *age* and *gender* needed to be controlled for in the main analyses (see Table 1). Results indicated that *gender* significantly positively correlated with anxiety/depression and prosocial behavior, but it correlated negatively with emotion regulation. In addition, *age* correlated positively with *emotion regulation*. Consequently, *age* and *experienced discrimination* were centered to account for multicollinearity, and interaction terms between *age* × *experienced discrimination*, and *gender* × *experienced discrimination* were created. The same process was done for *expected discrimination*. No significant interactions were detected for any of the four emotional state measures. Consequently, the reported main results are based on the regression analyses conducted without the hypothesized interaction terms in each model.

## Results

### Internalizing Problems

#### Anxiety/Depression

First, a hierarchical linear regression tested whether *age*, *gender*, and *experienced discrimination* were uniquely associated with higher *anxiety/depression* scores. Results showed that the model significantly accounted for 12.25% of the variance in outcome scores, [*F* (5, 1121) = 31.31, *p* < 0.001] (Table 2). *Gender* [*B* = 1.86, *p* < 0.001] and *experienced discrimination* [*B* = 0.12, *p* = 0.013] were significantly associated with anxiety/depression in Black youth. In other words, anxiety/depression was significantly higher for girls than for boys, and as experienced discrimination increased for Black youth, so did anxiety and depression.

Next, another hierarchical linear regression tested whether *age*, *gender*, and *expected discrimination* were uniquely associated with higher *anxiety/depression* scores. This model was also significant and accounted for 12.20% of the variance in outcome scores, [*F*(5, 1120) = 30.99, *p* < 0.001] (Table 3). Similar patterns were found to the model with *expected discrimination* in that both *gender* [*B* = 1.86, *p* < 0.001] and *expected discrimination* [*B* = 0.15, *p* = 0.036] were significantly associated with anxiety/depression.

Finally, a third hierarchical linear regression combined both discrimination variables into the same model and step, along with age and gender, to test whether one form of discrimination predicted anxiety/depression more than the other. This model was also significant and accounted for 12.42% of the variance in outcome scores, [*F*(6, 1114) = 26.33, *p* < 0.001] (Table 4). While *gender* [*B* = 1.87, *p* < 0.001] was still significantly associated, neither *experienced* nor *expected discrimination* significantly contributed when both were added into the model.

#### Suicidal Thoughts

Hierarchical binary logistic regression was used to test associations between independent variables and suicidal thoughts. The overall model was found to be statistically significant (*χ*^2^ (4) = 14.04, *p* = 0.007), with Nagelkerke’s *R*^2^ value of 0.04. Age [*χ*^2^ (1) = 1.20, *p* = 0.274, *OR* = 1.12, 95% *CI* = 0.91–1.37] was not statistically significant. However, *gender* [*χ*^2^ (1) = 5.15, *p* = 0.023, *OR* = 1.72, 95% *CI* = 1.08–1.37] and *experienced discrimination* [*χ*^2^ (1) = 7.78, *p* = 0.005, *OR* = 1.21, 95% *CI* = 1.06 – 1.39] were found to be statistically significant in predicting the odds of suicide ideation.

When exchanging *experienced discrimination* with *expected discrimination*, similar results were found again. The model was found to be statistically significant (*χ*^2^ (4) = 26.80, *p* < 0.001), with Nagelkerke’s *R*^2^ value of 0.08. Age [*χ*^2^ (1) = 0.53, *p* = 0.466, *OR* = 1.08, 95% *CI* = 0.88–1.32] was not statistically significant. However, *gender* [*χ*^2^ (1) = 5.76, *p* = 0.016, *OR* = 1.80, 95% *CI* = 1.11–2.89] and *expected discrimination (χ*^2^ (1) = 18.50, *p* < 0.001, *OR* = 1.62, 95% *CI* = 1.30–2.02) were found to be statistically significant in predicting the odds of suicide ideation.

Then, when combining *experienced* and *expected discrimination* in the same model, the impact of the variables diverged. The model was found to be statistically significant (*χ*^2^ (5) = 27.70, *p* < 0.001), with Nagelkerke’s *R*^2^ value of 0.09. Age [*χ*^2^ (1) = 0.49, *p* = 0.486, *OR* = 1.08, 95% *CI* = 0.88–1.32] was not statistically significant. However, *gender* [*χ*^2^ (1) = 6.20, *p* = 0.013, *OR* = 1.84, 95% *CI* = 1.14–2.89] and *expected discrimination* [*χ*^2^ (1) = 12.82, *p* < 0.001, *OR* = 1.54, 95% *CI* = 1.22–1.95] were statistically significant, but *experienced discrimination* was not.

### Social-Emotional Development

#### Prosocial Behavior

*Age*, *gender,* and *experienced discrimination* accounted for 4.31% of the variance in prosocial behavior scores in a hierarchical linear regression, [*F*(5, 1124) = 10.31, *p* < 0.001]. Only *gender* was associated with prosocial behavior [*B* = 1.02, *p* < 0.001].

However, the patterns changed with *expected discrimination.* The model accounted for 4.74% of the variance in prosocial behavior scores [*F*(5, 1123) = 11.13, *p* < 0.001] with both *gender* [*B* = 0.99, *p* < 0.001] and *expected discrimination* [*B* =  −0.0.20, *p* = 0.004] significantly contributing to the variance in those scores.

When both experienced and expected discrimination were added in the same model in the same step, the same patterns emerged as in the suicide ideation model. The model accounted for 4.80% of the variance in prosocial behavior scores [*F*(6, 1117) = 9.39, *p* < 0.001] with both *gender* [*B* = 0.99, *p* < 0.001] and *expected discrimination* [*B* =  −0.0.17, *p* = 0.020] significantly contributing to the variance in those scores, but not *experienced discrimination*.

#### Emotion Regulation

In the final set of hierarchical linear regressions, the first model significantly accounted for 4.00% of variance in emotion regulation scores, [*F*(5, 1121) = 9.29, *p* < 0.001]. All three variables were uniquely and significantly associated with emotion regulation scores. *Age* [*B* = 0.17, *p* < 0.028], *gender* [*B* =  −1.01, *p* < 0.001], and *experienced discrimination* significantly associated with emotion regulation [*B* =  −0.20, *p* < 0.001].

When *expected discrimination* was swapped into the model, the second model accounted for 5.00% of emotion regulation scores [*F*(5, 1115) = 11.62, *p* < 0.001]. All three predictors including *age* [*B* = 0.15, *p* = 0.047], *gender* [*B* =  −1.06, *p* < 0.001], and *expected discrimination* [*B* =  −0.37, *p* < 0.001] significantly explained outcome scores with similar patterns.

Finally, when both *experienced* and *expected* discrimination were added into the model, the model accounted for 5.33% of emotion regulation scores [*F*(6, 1114) = 10.46, *p* < 0.001]. All four predictors, namely, *age* [*B* = 0.17, *p* = 0.029], *gender* [*B* =  −1.08, *p* < 0.001], *experienced discrimination* [*B* =  −0.13, *p* < 0.034], and *expected discrimination* [*B* =  −0.30, *p* < 0.001], significantly explained outcome scores.

## Discussion

This study aimed to replicate and expand prior research that connected health and wellness outcomes with experiences of racial discrimination in Black youth (Kirkinis et al., 2018; Seaton & Yip, 2009; Seaton et al., 2010). The first goal of this investigation was to reexamine the association between racial discrimination and internalizing problems. In line with previous research (e.g., Seaton & Yip, 2009), these data suggest that experienced racial discrimination had small effects (Sullivan & Feinn, 2012) positively associated with anxiety/depression for Black youth. This paper also aimed to retest for a positive association between experienced racial discrimination and suicidal thoughts. Though the sample size for this item was lower than the others because one community excluded the item from their survey, descriptive statistics were in line with national trends (Lindsey et al., 2019); around 18% of youth had suicidal thoughts in the past 12 months. Results found that the more often youth experienced racial discrimination, the more likely they were to contemplate suicide. Though the effect of racial discrimination on suicidal thoughts was small (Sullivan & Feinn, 2012), the data reaffirmed previous research on Black youth (Walker et al., 2017).

Investigations on the association between these experiences and mental health have primarily focused on negative emotional states. However, the second goal of this investigation was to add to the literature by understanding the association between experienced racial discrimination and social-emotional development. This was achieved by examining the association of experienced racial discrimination with prosocial behavior and emotion regulation. Prosocial behavior (Raposa et al., 2016) and emotion regulation (Troy & Mauss, 2011) can serve as protective factors or a skillset to ameliorate challenges encountered in life which can ultimately lead to better health outcomes. This was the first study of its kind to examine the relationship between racial discrimination and prosocial development in both Black boys and Black girls. Results found a negative association between experienced racial discrimination and emotion regulation but no significant relationship between experienced racial discrimination and prosocial behavior. The finding on prosocial behavior thus neither supports the hypothesis nor previous research with male adolescents (Lozada et al., 2017).

The current study also examined whether the patterns for expected discrimination were similar to experienced discrimination. The relationship between thoughts on how race can impact future opportunities and youth well-being is not well understood. It is important to highlight in the results that a much higher proportion of youth expected that their race would stop them from getting ahead in life in the future (71.11%) in contrast to those who experienced discrimination (31.57–45.10%). In regression analyses, experienced discrimination and expectations around discrimination did not produce the same patterns when combined into a model. When it came to anxiety and depression, the two forms of discrimination appeared to cancel each other out and account for the same variance in explaining the outcome. However, when it came to suicide ideation and prosocial behavior, only expectations around discrimination explained the outcomes. Finally, for emotion regulation, both forms of discrimination explained the outcome scores, but expected discrimination had a greater association with the outcome. Thus, what these results shed light on is that it may in fact be valuable to separate out different forms of discrimination to understand the impact they have on youth outcomes. It is interesting that while experiences and expectations explained anxiety and depression in a similar way, expectations were particularly important for other youth outcomes, like emotion regulation, which may question the long-term effects of discrimination in explaining youths emotions and their interactions with others.

Lastly, this study explored how age and gender moderated the relationship between racial discrimination and emotional states in Black youth. While no significant interactions were detected, there were main effects for both age and gender. In line with previous research, which shows that emotion regulation increases during adolescence (Zimmerman & Iwanski, 2014), age was positively associated with emotion regulation. It is possible that there were no interactions for age because there was not a wide enough range to detect age-related differences.

Main effects for gender were detected in all analyses. Specifically, scores were higher for Black girls than Black boys on every measure except emotion regulation. The lack of significant interactions between gender and either experienced or expected racial discrimination suggests that discrimination may not impact boys and girls differently, which is counter to what is presented in the news (e.g., Badger et al., 2018) and in research (e.g., Kwate & Goodman, 2015) that discrimination is experienced by boys more.

### Limitations

These findings need to be considered in the context of some limitations. First, could be the use of single-item assessment. Both suicidal thoughts and expected discrimination were captured with a single item. The value in a single-item assessment is that it permits more time to capture other constructs and can reduce the chances of fatigue on participants. Additionally, research supports that many single-item assessments are in agreement with their multi-item subscales (Verster et al., 2021). However, according to Verster and colleagues, single items do not often have good agreement with full scales that tend to by multifaceted with several subscales. Additionally, they are not ideal in situations of diagnosing patients. For instance, one study found that a single item on suicide ideation can overestimate those truly experiencing suicidal thoughts by 8% (Millner et al., 2015). However, given that this study was not designed for patient intervention, having a single item capture suicide ideation is less of a concern.

A third limitation is the cross-sectional nature of the design. A longitudinal design would have permitted the test in a mediation model to understand how something such as social-emotional development could mediate the relationship between different forms of discrimination and poor mental health outcomes. The use of a cross-sectional design could have obscured the direction of effects implied by the regression analyses. For instance, while the results support that expected discrimination could be detrimental for both internalizing problems and social-emotional development it is also possible that internalizing problems and challenges with social-emotional development might increase the concern that one’s race might interfere in their future.

### Implications for Community Prevention Systems

Data from this study support that racial discrimination is a risk factor not only for poor mental health outcomes like suicidal thoughts, but it also impacts protective factors around social-emotional development that mitigate negative daily experiences. Consequently, this study validates the importance of any needs assessment or epidemiological survey examining a broad range of risks/protection/and outcomes. For instance, the website Blueprints for Healthy Youth and Development (https://www.blueprintsprograms.org/) was broadened from focusing only on violence prevention to five outcome domains: problem behavior, education, physical health, emotional well-being, and positive relationships. The website organizes information such that it specifies which risk and protective factors are associated with these outcomes, highlighting the importance of this approach. Therefore, programs addressing racial discrimination should consider how risk and protective factors can relate when addressing youth mental health.

These findings also reinforce the importance in addressing systemic racism. Youth from the current study were from majority Black communities which could in part explain why youth were less optimistic about their future than those who experienced discrimination first hand. Yet, despite this, there still appears to be a consistently negative impact of racial discrimination on a range of outcomes. Future studies should focus on communities where Black youth are not the largest minority group to better understand how discrimination impact mental health outcomes. While evidence-based programs could use a risk and protective factor approach to improve youth outcomes, it is best to address the root cause: racial discrimination in the system. Brody et al. (2006) suggest that “those at the receiving end of discrimination can, overtime, come to internalize the discrimination view” (p. 1183). In addition, since the context in which these discriminatory experiences occurred was not asked about, it is challenging to know if youth in this study experienced discrimination in community settings, such as retail stores or schools, where they are likely to encountered non-Black personnel. However, regardless of which systems experiences took place, the data from this study demonstrates that expectations about systemic racism can have an equally detrimental impact on adolescents’ mental well-being.

The push for equitable services must continue to persist. It is impossible to predict the magnitude that eliminating discriminatory experiences for Black youth could have on their mental health and life trajectory. It could begin with youth connecting more to their community or having increased self-worth. However, in the long term, it may lead to reduced incarceration, homelessness, comorbidities, and even Medicare spending.

### Future Directions

These findings lend to future considerations around measurement quality, moderators, and the developmental impact of discrimination. The frequency and intensity of racially motivated experiences is only going to be detected to the extent that the measure tolerates. In a meta-analysis on the impact of discrimination on well-being during adolescence, 40 measures on racial discrimination were used (Benner et al., 2018). Therefore, just as it was suggested in Benner et al., more measurement work is still needed to understand the complexity of racial discrimination (e.g., timing, perpetrator, experienced vs. witnessed). For instance, the impact may be different whether these interactions are with peers, administrators, sales associates, or law enforcement. Future research on this topic should also employ cross-sectional and longitudinal designs. These types of designs could elucidate how events (e.g., Brianna Taylor) impact youth both in the immediate and long terms, but also to better understand the cumulative effect of perceived systematic discriminatory incidents in the lived experiences of school-aged youth. These approaches would establish developmental approaches that better serve younger children, younger adolescents, older adolescents, and youth transitioning to adulthood in dealing with the deleterious impacts of discrimination on well-being and identity. In addition, more research is needed on how experiences of discrimination shape youths’ cognitions about their self-efficacy so that it can be addressed in interventions until health equity is created.

Research needs to investigate the impact of racial discrimination over the lifespan. In other words, are there differential outcomes for youth who report lower levels of racial discrimination during the adolescent years compared to youth that report higher levels of racial discrimination? Similarly, it should not be assumed that the types of racial discrimination experienced during adolescence are the same experienced as youth transition to adulthood or in stages of adulthood. Racial discrimination in public schools may be qualitatively different than those experiences at college/university or the workforce. Longitudinal research may facilitate an understanding, and this work needs to involve both quantitative and qualitative approaches to better understand the life impact of discrimination for the Black community.

## Data Availability

If you would like access to this data, we will work with our institutions and the communities involved in data collection to make the data as available as we can with appropriate protections.

## Appendix. Proportion of Black Adolescents who Experienced Discrimination

**Table:**

	**Sample size**	**Participant age (years)**	**Scale name**	**Length of reflection**	**Proportion**
Sellers et al. (2006)	314	*M* = 13.8; range = 11–17	Daily Life Experiences Scale	Past 12 months	62.5%
Seaton et al. (2008)	810	*M* = 15; range = 13–17	Everyday Discrimination Scale	Past 12 months	87%
Dulin-Keita et al. (2011)	57	M = 9.72; range = 7–12	Williams Every-Day Discrimination Scale (racial discrimination subscale)	Past 30 days	19.99%
Roberts et al. (2012)	745	*M* = 10.5; range = 10–11	Schedule of Racist Events	General experiences	38% (more than minimal experience); 89% (some experience)
McNeil Smith and Fincham (2015)	711	*M* = 14.24; range = 13–17	School Discrimination Scale	General experiences	22–40%
Nagata et al. (2021)	1656	*M* = N/A; range 10–11	Perceived Discrimination Scale	General experiences	10%
Current Paper	1156	*M* = 16.57; range = 14–19	Experienced Discrimination; Expected Discrimination	Past 12 months; general experiences	Experienced (48.83%); expected (71.11%)

## References

[CR1] Annie E. Casey Foundation (2023). *Evidence2Success: Involving communities in assessing and improving the well-being of children and young people.* https://www.aecf.org/work/community-change/evidence2success/

[CR2] Assari, S., Mistry, R., Lee, D. B., Caldwell, C. H., & Zimmerman, M. A. (2019). Perceived racial discrimination and marijuana use a decade later; Gender differences among black youth. *Frontiers in Pediatrics,**7*(78), 1–11. 10.3389/fped.2019.00078 30968004 10.3389/fped.2019.00078 PMC6438901

[CR3] Badger, E., Miller, C. C., Pearce, A., & Quealy, K. (2018). Extensive data shows punishing reach of racism for black boys. *New York Times*, *19*.

[CR4] Benner, A. D., Wang, Y., Shen, Y., Boyle, A. E., Polk, R., & Cheng, Y. P. (2018). Racial/ethnic discrimination and well-being during adolescence: A meta-analytic review. *American Psychologist,**73*(7), 855–883. 10.1037/amp0000204 30024216 10.1037/amp0000204 PMC6172152

[CR5] Brody, G. H., Chen, Y. F., Murry, V. M., Ge, X., Simons, R. L., Gibbons, F. X., Gerrard, M., & Cutrona, C. E. (2006). Perceived discrimination and the adjustment of African American youths: A five-year longitudinal analysis with contextual moderation effects. *Child Development,**77*(5), 1170–1189. 10.1111/j.1467-8624.2006.00927.x 16999791 10.1111/j.1467-8624.2006.00927.x

[CR6] Copping, K. E., Jurtz-Costes, B., Rowley, S. J., & Wood, D. (2013). Age and race differences in racial stereotype awareness and endorsement. *Journal of Applied Social Psychology,**43*(5), 971–980. 10.1111/jasp.12061 23729837 10.1111/jasp.12061 PMC3667990

[CR7] Cunningham, M., Mulser, R. M., Scott, K., & Yates, A. (2019). African American adolescents speak: the meaning of racial identity in the relation between individual race-related stress and depressive symptoms. In H. E. Fitzgerald, D. J. Johnson, D. B. Qin, F. A. Villarruel, & J. Norder (Eds.), *Handbook of children and prejudice: integrating research, practice, and policy. *Switzerland: Springer. 10.1007/978-3-030-12228-7_30

[CR8] Davis, A. N., McGinley, M., Carlo, G., Schwartz, S. J., Unger, J. B., Rosiers, S. E. D., Baezconde-Garbanati, L., Lorenzo-Blanco, E. I., & Soto, D. (2021). Examining discrimination and familism values as longitudinal predictors of prosocial behaviors among recent immigrant adolescents. *International Journal of Behavioral Development,**45*(4), 317–326. 10.1177/01650254211005561 39005848 10.1177/01650254211005561 PMC11245281

[CR9] Davis, A. N., & Carlo, G. (2019). Toward an integrative conceptual model on the relations between discrimination and prosocial behaviors in US Latino/Latina youth. *Handbook of children and prejudice* (pp. 375–388). Cham: Springer.

[CR10] Dulin-Keita, A., Hannon Iii, L., Fernandez, J. R., & Cockerham, W. C. (2011). The defining moment: Children’s conceptualization of race and experiences with racial discrimination. *Ethnic and Racial Studies,**34*(4), 662–682. 10.1080/01419870.2011.535906 21532908 10.1080/01419870.2011.535906 PMC3083924

[CR11] Herda, D. (2016). The specter of discrimination: Fear of interpersonal racial discrimination among adolescents in Chicago. *Social Science Research,**55*, 48–62. 10.1016/j.ssresearch.2015.09.010 26680287 10.1016/j.ssresearch.2015.09.010

[CR12] International Youth Development Study (IYDS) Student Survey (2003). Social Development Research Group, University of Washington, Seattle. http://www.sdrg.org/current.asp

[CR13] Kirkinis, K., Pieterse, A. L., Martin, C., Agiliga, A., & Brownell, A. (2018). Racism, racial discrimination, and trauma: A systematic review of the social science literature. *Ethnicity and Health,**45*, 232–259. 10.1080/13557858.2018.1514453 10.1080/13557858.2018.1514453 30165756

[CR14] Kwate, N. O. A., & Goodman, M. S. (2015). Racism at the intersections: gender and socioeconomic differences in the experience of racism among African Americans. *American Journal of Orthopsychiatry,**85*(5), 397–408. 10.1037/ort0000086 26460700 10.1037/ort0000086

[CR15] Lindsey, M. A., Sheftall, A. H., Xiao, Y., & Joe, S. (2019). Trends of suicidal behaviors among high school students in the United States: 1991–2017. *Pediatrics*, *144*(5). 10.1542/peds.2019-1187 10.1542/peds.2019-1187 PMC7299440 31611338

[CR16] Lozada, F. T., Jagers, R. J., Smith, C. D., Bañales, J., & Hope, E. C. (2017). Prosocial behaviors of Black adolescent boys: An application of a sociopolitical development theory. *Journal of Black Psychology,**43*(5), 493–516. 10.1177/0095798416652021 10.1177/0095798416652021

[CR17] McNeil Smith, S., & Fincham, F. (2015). Racial discrimination experiences among black youth. *Journal of Black Psychology,**42*(4), 300–319. 10.1177/0095798415573315 10.1177/0095798415573315

[CR18] Millner, A. J., Lee, M. D., & Nock, M. K. (2015). Single-item measurement of suicidal behaviors: Validity and consequences of misclassification. *PLoS ONE,**10*(10), 1–17. 10.1371/journal.pone.0141606 10.1371/journal.pone.0141606 PMC4619664 26496707

[CR19] Nagata, J. M., Ganson, K. T., Sajjad, O. M., Benabou, S. E., & Bibbins-Domingo, K. (2021). Prevalence of perceived racism and discrimination among US children aged 10 and 11 years: The adolescent brain cognitive development (ABCD) Study. *JAMA pediatrics*. 10.1001/jamapediatrics.2021.1022 10.1001/jamapediatrics.2021.1022 PMC8129899 33999104

[CR20] Padilla-Walker, L. M., Carlo, G., Christensen, K. J., & Yorgason, J. B. (2012). Bidirectional relations between authoritative parenting and adolescents’ prosocial behaviors. *Journal of Research on Adolescence,**22*(3), 400–408. 10.1111/j.1532-7795.2012.00807.x 10.1111/j.1532-7795.2012.00807.x

[CR21] Raposa, E. B., Laws, H. B., & Ansell, E. B. (2016). Prosocial behavior mitigates the negative effects of stress in everyday life. *Clinical Psychological Science,**4*(4), 691–698. 10.1177/2167702615611073 27500075 10.1177/2167702615611073 PMC4974016

[CR22] Riley, T. N., DeLaney, E., Brown, D., Lozada, F. T., Williams, C. D., & Dick, D. M. (2021). The associations between African American emerging adults’ racial discrimination and civic engagement via emotion regulation. *Cultural Diversity and Ethnic Minority Psychology,**27*(2), 169. 10.1037/cdp0000335 32281807 10.1037/cdp0000335

[CR23] Roberts, M. E., Gibbons, F. X., Gerrard, M., Weng, C.-Y., Murry, V. M., Simons, L. G., Simons, R. L., & Lorenz, F. O. (2012). From racial discrimination to risky sex: Prospective relations involving peers and parents. *Developmental Psychology,**48*(1), 89–102. 10.1037/a0025430 21942666 10.1037/a0025430 PMC3807851

[CR24] Seaton, E. K., & Yip, T. (2009). School and neighborhood contexts, perceptions of racial discrimination, and psychological well-being among African American adolescents. *Journal of Youth and Adolescence,**38*(2), 153–163. 10.1007/s10964-008-9356-x 19636714 10.1007/s10964-008-9356-x PMC4574483

[CR25] Seaton, E. K., Caldwell, C. H., Sellers, R. M., & Jackson, J. S. (2008). The prevalence of perceived discrimination amongAfrican American and Caribbean Black youth. *Developmental Psychology,**44*(5), 1288–1297. 10.1037/a0012747 18793063 10.1037/a0012747 PMC2556985

[CR26] Seaton, E. K., Caldwell, C. H., Sellers, R. M., & Jackson, J. S. (2010). An intersectional approach for understanding perceived discrimination and psychological well-being among African American and Caribbean black youth. *Developmental Psychology,**46*(5), 1372–1379. 10.1037/a0019869 20822246 10.1037/a0019869 PMC3755457

[CR27] Sellers, R. M., Linder, N. C., Martin, P. M., & Lewis, R. L. (2006). Racial identity matters: The relation between racial discrimination and psychological functioning in African American adolescents. *Journal of Research on Adolescence,**16*(2), 187–216. 10.1111/j.1532-7795.2006.00128.x 10.1111/j.1532-7795.2006.00128.x

[CR28] Sullivan, G. M., & Feinn, R. (2012). Using effect size—or why the P value is not enough. *Journal of Graduate Medical Education,**4*(3), 279–282. 10.4300/JGME-D-12-00156.1 23997866 10.4300/JGME-D-12-00156.1 PMC3444174

[CR29] Taylor, R. D., Oberle, E., Durlak, J. A., & Weissberg, R. P. (2017). Promoting positive youth development through school-based social and emotional learning interventions: A meta-analysis of follow-up effects. *Child Development,**88*(4), 1156–1171. 10.1111/cdev.12864 28685826 10.1111/cdev.12864

[CR30] Troy, A. S., & Mauss, I. B. (2011). Resilience in the face of stress: Emotion regulation as a protective factor. In S. M. Southwick, B. T. Litz, D. Charney, & M. Friedman (Eds.), *Resilience and Mental Health: Challenges across the Lifespan* (pp. 30–44). Cambridge University Press.

[CR31] Umaña-Taylor, A. J. (2016). A post-racial society in which ethnic-racial discrimination still exists and has significant consequences for youths’ adjustment. *Current Directions in Psychological Science,**25*(2), 111–118. 10.1177/0963721415627858 10.1177/0963721415627858

[CR32] Verster, J. C., Sandalova, E., Garssen, J., & Bruce, G. (2021). The use of single-item ratings versus traditional multiple-item questionnaires to assess mood and health. *European Journal of Investigation in Health, Psychology and Education,**11*(1), 183–198. 10.3390/ejihpe11010015 34542458 10.3390/ejihpe11010015 PMC8314344

[CR33] Walker, R. L., Salami, T. K., Carter, S. E., & Flowers, K. (2014). Perceived racism and suicide ideation: Mediating role of depression but moderating role of religiosity among African American adults. *Suicide and Life-Threatening Behavior,**44*(5), 548–559. 10.1111/sltb.12089 24690042 10.1111/sltb.12089

[CR34] Walker, R., Francis, D., Brody, G., Simons, R., Cutrona, C., & Gibbons, F. (2017). A longitudinal study of racial discrimination and risk for death ideation in African American youth. *Suicide and Life-Threatening Behavior,**47*(1), 86–102. 10.1111/sltb.12251 27137139 10.1111/sltb.12251

[CR35] Williams, D. R., Lawrence, J. A., & Davis, B. A. (2019). Racism and health: Evidence and needed research. *Annual Review of Public Health,**40*, 105–125. 10.1146/annurev-publhealth-040218-043750 30601726 10.1146/annurev-publhealth-040218-043750 PMC6532402

[CR36] Zimmermann, P., & Iwanski, A. (2014). Emotion regulation from early adolescence to emerging adulthood and middle adulthood: Age differences, gender differences, and emotion-specific developmental variations. *International Journal of Behavioral Development,**38*(2), 182–194. 10.1177/0165025413515405 10.1177/0165025413515405

[CR37] Zimmerman, G. M., & Miller-Smith, A. (2021). The impact of anticipated, vicarious, and experienced racial and ethnic discrimination on depression and suicidal behavior among Chicago youth. *Social Science Research*, 102623. 10.1016/j.ssresearch.2021.102623 10.1016/j.ssresearch.2021.102623 34823672

